# Die Promotionsordnungen der medizinischen Fakultäten in Deutschland: Was hat sich in den letzten 9 Jahren verändert?

**DOI:** 10.1007/s00104-025-02387-9

**Published:** 2025-10-02

**Authors:** Tomico van Bergen, Christoph Paasch, Richard Hunger, René Mantke

**Affiliations:** 1 Fakultät für Medizin, Medizinische Hochschule Brandenburg Theodor Fontane, Brandenburg, Deutschland; 2 Klinik für Allgemein- und Viszeralchirurgie, Universitätsklinikum Brandenburg an der Havel, Hochstr. 29, 14770 Brandenburg an der Havel, Deutschland

**Keywords:** Promotion, Dissertation, Betreuungsvereinbarung, MFT, Wissenschaftsrat, Doctorate, Dissertation, Supervision agreement, MFT, German Science and Humanities Council

## Abstract

**Hintergrund:**

Die Promotionsordnungen an deutschen Fakultäten weisen teils grundlegende Unterschiede auf. Vor diesem Hintergrund wurde ein Positionspapier des Medizinischen Fakultätentages (MFT) zur Vermittlung von Wissenschaftskompetenz im Medizinstudium im Jahr 2016 verfasst. Das vorliegende Studienprojekt soll nun 9 Jahre später die Qualität der Promotionsordnungen in Deutschland untersuchen.

**Methoden:**

Es wurden die 39 aktuellen Promotionsordnungen (PromO) aller deutschen medizinischen Fakultäten mit Promotionsrecht herangezogen und analysiert. Zusätzlich wurden die Betreuungsvereinbarungen, falls vorhanden, aus den gleichen Quellen miteinbezogen. Der Anspruch der Satzungen wurde systematisch gemäß dem Scoringsystem nach Sorg et al. bewertet.

**Ergebnisse:**

Der Mittelwert der Gesamtpunktzahl aller Promotionsordnungen beträgt 68,4 Punkte (SD: ± 8,4) und hat sich im Vergleich zu 2016 (57,5 Punkte, SD: ± 9,4) signifikant erhöht (*p* < 0,001). Die Verpflichtung zum Abschluss einer Betreuungsvereinbarung und die Möglichkeit der kumulativen Promotion wurden seit 2016 stark kodifiziert. Die Einführung in Methodenkenntnisse und die Überprüfung auf Plagiate sind im Vergleich zu 2016 vermehrt vorgeschrieben, aber nicht flächendeckend verbreitet. Die vom MFT geforderten mindestens 9 Monate andauernde Forschungstätigkeit wurde nur von einer medizinischen Fakultät adressiert. Nicht alle Promotionen müssen publiziert werden.

**Schlussfolgerung:**

Die Kernpunkte des MFT-Positionspapiers wurden nicht vollständig umgesetzt. Die Qualität der Promotionsordnungen in Deutschland hat sich in den letzten 9 Jahren jedoch verbessert.

**Graphic abstract:**

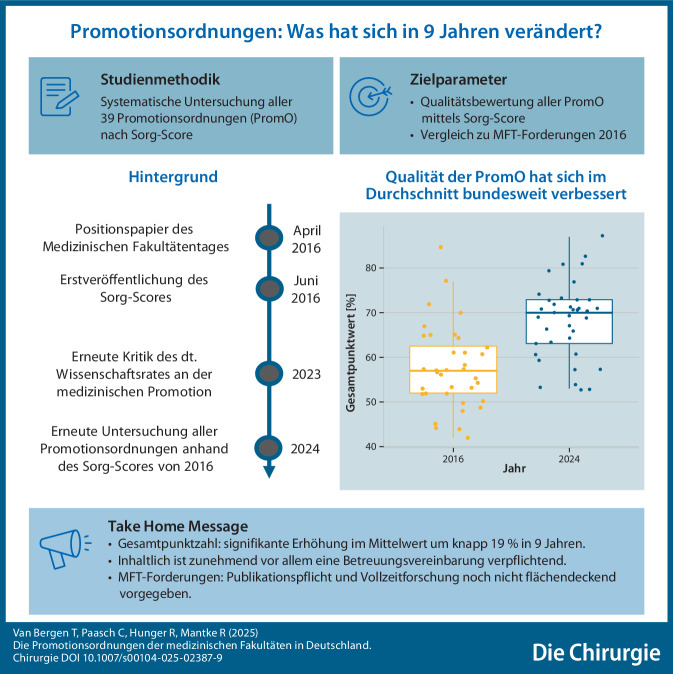

**Zusatzmaterial online:**

Die Online-Version dieses Beitrags (10.1007/s00104-025-02387-9) enthält eine weitere Tabelle mit der Darstellung der Score-Struktur nach Sorg et al.

## Abstract (en)

**Background:**

The doctoral degree regulations at German faculties partially show fundamental differences. Against this background in 2016 the Medical Faculty Association (MFT) published a position paper on teaching scientific competence in medical studies. This study project aims to analyze the quality of doctoral degree regulations in Germany 9 years later.

**Methods:**

In this study 39 current doctoral degree regulations (PromO) from German medical faculties with the right to award doctorates were analyzed along with the supervision agreements, if available. The standard of the statutes was assessed according to the scoring system of Sorg et al.

**Results:**

The mean value of the total score of all doctoral regulations was 68.4 points (SD ± 8.4) and has increased compared to 2016 (57.5 points, SD ± 9.4, *p* < 0.001). Supervision agreements and cumulative doctorates have been strongly codified since 2016. Methodological training and plagiarism checks have become more mandatory compared to 2016 but are not widespread. The MFT requirement of at least 9 months of research activity was only addressed by 1 medical faculty. Not all doctorates have to be published.

**Conclusion:**

The key points of the MFT position paper have not been fully implemented; however, the quality of doctoral regulations in Germany has improved over the last 9 years.

Die Promotion zum Dr. med. ist für viele Studierende/Ärzte ein wichtiger Meilenstein der akademischen Ausbildung. Umso bemerkenswerter ist es, dass sich Anforderungen und Struktur der verschiedenen Promotionsordnungen mitunter erheblich unterscheiden.

Die Promotionspraxis in Deutschland im Fachbereich Medizin wird aus diversen Gründen, insbesondere jedoch aufgrund der häufig studienbegleitenden Promotionsphase u. a. vom Wissenschaftsrat wiederholt kritisiert. Der European Research Council (ERC) geht sogar so weit, dass er die deutsche Medizin-Promotion überhaupt „nicht als Nachweis selbstständiger Forschung“ anerkennt [2].

Von zentraler Bedeutung für diese Arbeit ist daher das Positionspapier des Medizinischen Fakultätentages (MFT) zur Vermittlung von Wissenschaftskompetenz im Medizinstudium aus dem Jahr 2016 [3]. In diesem Positionspapier des Verbands der medizinischen Ausbildungs- und Forschungsstätten in Deutschland werden konkrete Vorschläge zur Förderung der wissenschaftlichen Ausbildung von Medizinstudierenden gemacht, welche durch z. B. die Einführung einer „mindestens neunmonatige[n] ganztägige[n] Tätigkeit“ ([2, 3], S. 29) auch Auswirkungen auf die Promotionsstrukturen haben könnten.

Kurze Zeit später erschien eine quantitative Analyse der Promotionsordnungen medizinischer Fakultäten, welche *große Unterschiede in Promotionsordnungen deutscher, medizinischer Fakultäten auf*zeigt [4]. Auch hier werden Vorschläge für eine Veränderung gemacht, beispielsweise eine „bundesdeutsche Rahmenverordnung für die medizinische Promotion“ [4].

Anfang 2024 wurde ein aktualisierter Vergleich der Promotionsordnungen in Deutschland durchgeführt und in englischer Sprache veröffentlicht [5]. Diese Erhebung erfolgte zwar nach der groben Struktur des Bewertungsscores von Sorg et al., wendete ihn aber nicht explizit an und bietet daher nur eine begrenzte Möglichkeit zum Vergleich der untersuchten Kriterien. Zusätzlich wurden bei dieser Studie keine Betreuungsvereinbarungen mit einbezogen, obwohl diese Vereinbarungen häufig die Verpflichtung zur „guten wissenschaftlichen Praxis“ (GWP) beinhalten und nicht nur den Umfang der Arbeit, sondern auch den Umfang der Betreuung festlegen. Diese Betreuungsvereinbarungen werden von den Promotionsordnungen häufig vorgegeben, werden aber nicht im Umfang definiert. Da der Abschluss der Betreuungsvereinbarung jedoch verpflichtend ist, sind die dort beschriebenen Ziele, Vorgaben und Leistungen implizit dennoch relevant.

Vor dem Hintergrund der dargestellten Kritik an der medizinischen Promotion aus verschiedenen Kreisen sind eine präzise Erhebung der Verpflichtung zur Einhaltung der GWP sowie eine konkrete Vergleichbarkeit mit der Vorstudie von entscheidender Bedeutung. Damit trägt die Studie nicht nur zur wissenschaftlichen Debatte über die Heterogenität medizinischer Promotionen in Deutschland bei, sondern liefert auch eine fundierte Datengrundlage für mögliche Reformbestrebungen und macht diese auch in deutscher Sprache zugänglich.

## Methoden

Zur Analyse wurden 39 Promotionsordnungen von 40 Fakultäten online abgerufen. Die Fakultät Mannheim verwendet die Promotionsordnung der Fakultät Heidelberg und wird daher nicht gesondert berücksichtigt. Die Datenerhebung fand im Zeitraum vom 01.10.2024 bis 31.01.2025 statt.

Zusätzlich wurden auch die Ausführungsbestimmungen und Bewertungsempfehlungen von Dissertationsschriften untersucht, um die Bewertungsgrundlagen der Fakultäten korrekt abbilden zu können. Diese befanden sich häufig im Anhang an die Promotionsordnungen, stellten aber auch eigene Dokumente dar, welche von den Fakultätswebsites bezogen wurden.

### Primäre und sekundäre Endpunkte

Der primäre Endpunkt war die Qualitätsbewertung aller Promotionsordnungen im Jahr 2025, welche standardisiert mittels des Scoringsystems von Sorg et al. [4] quantifiziert wurde.

Sekundärer Endpunkt war der Vergleich aller Gesamtpunktzahlen im Vergleich zum Jahr 2016 und die Anzahl der geänderten Promotionsordnung (PromO) seit der letzten Erhebung.

### Bewertungsschema

Da Promotionsordnungen von jeder Fakultät individuell formuliert werden, ist eine objektive Einordnung der relevanten Punkte essenziell. Um diese herstellen zu können und gleichzeitig eine Vergleichbarkeit mit vorangegangenen Studien zu ermöglichen, greift diese Arbeit auf den Bewertungsansatz einer im Jahr 2016 durchgeführten Untersuchung von Promotionsordnungen zurück. Das Score-System wurde von den Autoren der ursprünglichen Studie Sorg et al. [4] entwickelt und umfasst 12 Kriterien. Diese Kriterien wurden „insbesondere im Hinblick auf die Qualität und Anforderungen der Promotionsleistungen, zusammengestellt und entsprechend der Anzahl der Nennungen in eine Rangliste gebracht und bewertet“ [2]. Der Score ist in der Tab. 2 (Zusatzmaterial online) näher erläutert.

Der Score gliedert sich in 4 Bereiche: A (maximal 24 Punkte) für Zulassungsvoraussetzungen, B (maximal 26 Punkte) für die Dissertation, C (maximal 24 Punkte) für die mündliche Prüfung und D (maximal 26 Punkte) für Begutachtung und Bewertung. Insgesamt können 100 Punkte erreicht werden, wobei eine höhere Punktzahl einen höheren Präzisionsgrad der Regularien anzeigt und höhere Anforderungen an die PromO stellt.

Weiterhin wurde das Datum des Inkrafttretens der PO bestimmt und daraus das Alter der PO abgeleitet.

### Statistische Auswertung

Gemäß dem publizierten Scoring-Algorithmus [4] wurden die Scores der Subskalen und der Gesamtskala berechnet. Die deskriptive Analyse umfasste die Zusammenfassung der statistischen Kennwerte, wobei hierfür das arithmetische Mittel und die Standardabweichung berechnet wurden. Die Prüfung der Verteilungsform erfolgte grafisch per Histogramm und teststatistisch mittels Shapiro-Wilk-Test. Der Vergleich der PromO-Scorewerte zwischen den beiden Zeitpunkten erfolgte mittels gepaarten t‑Tests. Für die Berechnung der bivariaten Zusammenhänge zwischen den Scorewerten der beiden Zeitpunkte bzw. dem Alter der PromO und dem Scorewert wurde der Pearson-Korrelationskoeffizient berechnet.

Die Datenauswertung wurde mit R (Version 4.4.2, The R Software Foundation) durchgeführt. Alle statistischen Tests wurden zweiseitig zum Signifikanzniveau von 5 % durchgeführt.

## Ergebnisse

**Abb. 1:**
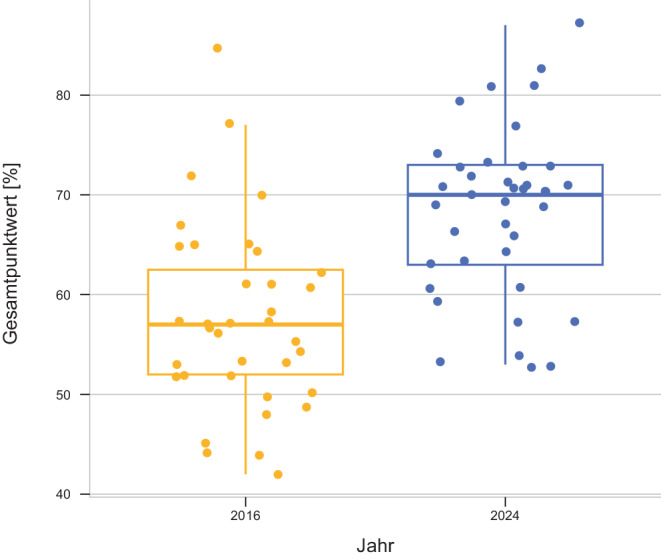
Darstellung der Gesamtbewertungen der Promotionsordnung mittels Box-Plot-Analyse (2016 *gelb*; 2024 *blau*). Der Mittelwert der Gesamtpunktzahl aller Promotionsordnungen beträgt 68,4 Punkte (SD: ± 8,4; Jahr 2024) und hat sich im Vergleich zu 2016 (57,5 Punkte, SD: ± 9,4) signifikant erhöht (*p* < 0,001)

Der Mittelwert der Gesamtpunktzahl aller Promotionsordnungen beträgt 68,4 Punkte (SD: ± 8,4; Jahr 2024) und hat sich im Vergleich zu 2016 (57,5 Punkte, SD: ± 9,4) signifikant erhöht (*p* < 0,001). Die Ergebnisse im Detail sind in Tab. 1; Abb. 1 und 2 dargestellt.

**Tab. 1:** Score-Ergebnisse der Promotionsordnungen in Deutschland

Fakultät	Teil A (0–24 Pkt.)	Teil B (0–26 Pkt.)	Teil C (0–24 Pkt.)	Teil D (0–26 Pkt.)	Gesamtergebnis (0–100 Pkt.)	Gesamt 2016 (0–100 Pkt.)
Brandenburg	20	23	18	26	87	*–*
Bielefeld	24	23	18	18	83	*–*
Hamburg	12	23	20	26	81	64
Lübeck	24	23	16	18	81	65
Köln	20	19	18	22	79	49
Düsseldorf	20	23	18	16	77	77
Würzburg	12	14	22	26	74	52
Duisburg-Essen	20	23	18	12	73	50
Gießen	16	23	18	16	73	67
Ulm	12	17	18	26	73	56
Witten-Herdecke	16	23	18	16	73	*–*
Erlangen-Nürnberg	12	26	22	12	72	62
Aachen	12	23	20	16	71	72
Berlin	16	23	18	14	71	85
Heidelberg	12	19	18	22	71	45
Marburg	12	23	18	18	71	48
Münster	8	19	20	24	71	61
Rostock	12	23	18	18	71	53
Hannover	12	16	20	22	70	57
Leipzig	16	20	18	16	70	58
Regensburg	16	16	22	16	70	50
Augsburg	24	19	16	10	69	*–*
Freiburg	16	23	22	8	69	53
München	20	19	16	14	69	55
Jena	8	23	20	16	67	61
TU München	16	20	16	14	66	70
Tübingen	12	20	16	18	66	52
Frankfurt	8	14	22	20	64	61
Bonn	16	23	16	8	63	44
Oldenburg	4	23	20	16	63	57
Dresden	8	23	18	12	61	65
Halle-Wittenberg	8	23	16	14	61	54
Homburg	8	17	20	14	59	44
Bochum	16	23	16	2	57	57
Göttingen	8	19	14	16	57	53
Greifswald	8	20	18	8	54	57
Kiel	8	13	20	12	53	52
Magdeburg	4	13	18	18	53	57
Mainz	12	23	12	6	53	42
*Mittelwert*	*13,5*	*20,5*	*18,3*	*16,1*	*68,4*	*57,3*
*Standardabweichung*	*5,3*	*3,4*	*2,3*	*5,7*	*8,4*	*9,4*

**Abb. 2:**
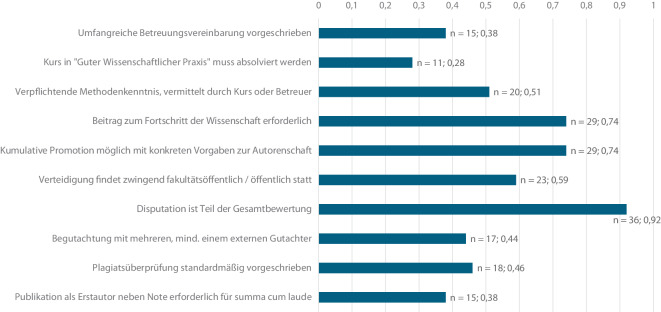
Die Abbildung gibt einen Überblick über den Anteil der Promotionsordnungen, die in der jeweiligen Kategorie den höchsten Anforderungen gerecht werden

### Teil A – Zulassungsvoraussetzungen (maximal 24 Punkte)

In der untersuchten Gruppe von Promotionsordnungen wurde bei 24 Fakultäten (62 %) eine schriftliche Vereinbarung zur Betreuung vorausgesetzt, in 15 PromO (38 %) wird eine umfangreiche Beschreibung inklusive Pflichten und Leistungen beider Unterzeichner gefordert.

Eine Verpflichtung auf GWP findet bei 26 Fakultäten (67 %) in der PromO oder der Betreuungsvereinbarung über eine schriftliche Verpflichtung statt. An 11 Fakultäten (28 %) ist der Abschluss eines Kurses mit dem Thema der GWP Voraussetzung für die Zulassung zur Promotion. Diese Kurse sind vereinzelt auch in strukturierte Promotionsprogramme eingebunden. An 2 Fakultäten gibt es diesbezüglich keine Verpflichtung.

Anders verhält es sich bei der Voraussetzung von Kursen der Methodik. Insgesamt 19 Fakultäten (49 %) nehmen in keinem Dokument Stellung zu einer solchen Verpflichtung. Im Gegensatz dazu wird in 11 PromO (28 %) der Nachweis über den Besuch eines Kurses in Methodik gefordert. Die Einführung in die wissenschaftliche Methodik soll gemäß 9 PromO (23 %) durch die Promotionsbetreuung erfolgen.

### Teil B – Dissertation (maximal 26 Punkte)

Insgesamt 29 PromO (74 %) setzen eine beachtliche wissenschaftliche Leistung oder einen Beitrag zum Fortschritt der Wissenschaft voraus.

In 35 Fakultäten (~90 %) sind Dissertationen auf Deutsch oder Englisch möglich, 3 erlauben weitere Sprachen auf Antrag. Nur Erlangen-Nürnberg fordert stets eine englische Zusammenfassung.

Hinsichtlich der Möglichkeiten einer kumulativen Promotion ergeben sich andere Zahlen als bei der Untersuchung 2016. Bei der damaligen Studie wurde „in 22 Promotionsordnungen eine genaue Beschreibung der kumulativen Dissertation gefunden“ [4]. In der aktuellen Untersuchung erhöht sich dieser Wert auf 29 PromO (74 %), in welchen Hinweisen auf Anzahl, Qualität des Journals und Autorenschaft gegeben werden. Fünf Fakultäten (13 %) bieten die Möglichkeit einer kumulativen Promotion, definieren aber keine Qualitätskriterien an die Publikationen. Vier PromO (10 %) geben Vorgaben für die Autorenschaft der Veröffentlichungen an. Die Universität Würzburg (3 %) bietet keine Möglichkeit der kumulativen Promotion in ihrer PromO.

### Teil C – mündliche Prüfungsleistung (maximal 24 Punkte)

Insgesamt 38 % (*n* = 15) der PromO prüfen neben der Dissertation das zugehörige Fachgebiet, 28 % (*n* = 11) nur das Fachwissen der Dissertation, weitere 28 % (*n* = 11) auch angrenzende Fachbereiche und 5 % (*n* = 2) zusätzlich die medizinischen Grundlagen der gesamten Medizin.

Bei der Beschreibung des zugelassenen Personenkreises der Verteidigung fordern 23 Fakultäten (59 %), die Disputation fakultätsöffentlich oder öffentlich abzuhalten; 16 PromO (41 %) nennen einen geschlossenen oder umschriebenen Personenkreis, welcher sich zumeist auf die Prüfungskommission beschränkt.

Weiterhin wurde untersucht, ob die Disputation benotet wird und inwieweit sie auf die Gesamtnote der Promotion Einfluss nimmt. Drei PromO setzen keine Benotung der Verteidigung voraus. Diese hat, abgesehen von der Bestehensgrenze, dadurch keinen Einfluss auf die Gesamtnote der Promotion.

### Teil D – Begutachtung und Bewertung (maximal 26 Punkte)

Die Begutachtung durch mehrere Gutachter regeln 17 PromO (44 %), wobei mindestens ein externer Gutachter in der Kommission beteiligt sein muss; 15 PromO (38 %) geben Vorgaben an, welche mit 6 Punkten bewertet werden. Sechs Fakultäten (15 %) geben eine Begutachtung ausschließlich durch interne Gutachter vor. Die Universität Bochum gibt in ihrer PromO (3 %) die Notwendigkeit der Begutachtung der Dissertation an, ohne weitere Details anzugeben.

Eine Heterogenität der Ergebnisse findet sich bei der Untersuchung auf Plagiatsüberprüfungen; 18 PromO (46 %) geben eine standardmäßige Überprüfung auf Plagiate an. Insgesamt 17 Fakultäten (44 %) geben keine Überprüfungen auf Plagiate an, 4 Fakultäten (10 %) führen vereinzelte Überprüfungen oder Prüfungen auf Verdacht in ihren PromO auf.

Das zwölfte Bewertungskriterium betrifft die Voraussetzungen für die Bewertung mit der Note „summa cum laude“. Bei 15 der PromO (38 %) ist das Kriterium erfüllt, wenn mindestens eine Publikation in einer Fachzeitschrift als Erstautor verfasst und eine mündliche Note mit der Bewertung „summa cum laude“ erlangt wurde.

## Diskussion

Am 13.04.2016 wurde das Positionspapier des medizinischen Fakultätentages (MFT) veröffentlicht. In diesem wurden Vorschläge zur Verbesserung der medizinischen Promotionen publiziert. Auf Grundlage dessen wurde von der Arbeitsgruppe Sorg im Juni 2016 eine Evaluierung der Promotionsordnungen durchgeführt und ein Bewertungsscore entwickelt [4]. Dieser wurde in der vorliegenden Arbeit 9 Jahre später erneut angewendet.

Diese Verbesserung beruht im Wesentlichen auf 2 Aspekten. Die Plagiatsüberprüfung ist nun häufiger (*n* = 18, 46 %) verpflichtend. Alle medizinischen Fakultäten haben eine Betreuungsvereinbarung als Grundlage des Promotionsverfahrens eingeführt.

### Anwendung von Plagiatsüberprüfungen

Ein zuletzt immer wichtiger gewordener Aspekt ist die Prüfung von Dissertationen auf Plagiate. Während die standardisierte Plagiatsprüfung bereits bei einigen Fakultäten stattfindet (*n* = 18; 46 %), gibt es vereinzelt nur Überprüfungen auf Verdacht (*n* = 4; 10 %). Häufig gibt es jedoch keine Angabe in den PromO auf solche Überprüfungen (*n* = 17; 44 %). Diese Anteile haben sich im Vergleich zur Erhebung von 2016 stark verbessert (damals waren an 6 Fakultäten vereinzelte Überprüfungen üblich [4]). Eine Auswirkung auf die Quote an Aberkennungen von verliehenen Graden müsste dabei noch untersucht werden.

Allerdings wird bei knapp der Hälfte der Fakultäten weiterhin kein Bezug genommen auf eine Überprüfung der Dissertationen auf Plagiate. Auch wenn die Anwendung von Plagiatssoftware *ihre eigenen Herausforderungen mit sich bringt *[9], wird es insbesondere mit Blick auf populäre Technologien wie Large Language Models (LLM) wie ChatGPT immer wichtiger, genaue Richtlinien für die Verwendung dieser zu veröffentlichen und eine entsprechende Einhaltung zu kontrollieren. Für diese Technologien „gibt es jedoch [aktuell] keine verlässliche Methode KI-generierte Texte nachzuweisen“ [10]. Die Fakultät der Universität Witten/Herdecke ist bislang die einzige Fakultät, welche in ihrer PromO Bezug zu diesem Thema nimmt und klare Regeln für die Verwendung vorgibt. Die Universität zu Köln veröffentlichte im Juli 2023 eine Übersichtsseite zu ChatGPT [10]. Im Sinne der Gleichwertigkeit von Promotionsbedingungen wäre es weiterhin dringend notwendig, wenn diese klaren Regeln auch an anderen Universitäten etabliert würden, um einheitliche Standards im Umgang mit neuen Technologien wie Large Language Models sicherzustellen und wissenschaftliches Fehlverhalten konsequent zu vermeiden.

### Umsetzung der Forderungen des MFT

Es stellt sich abschließend die Frage, in welchem Maße das MFT-Positionspapier über die letzten 9 Jahre umgesetzt wurde. Zu beachten ist hierbei, dass laut Statistik der Promovierenden zwischen 2019 und 2021 insgesamt 121.635 Promotionen abgeschlossen wurden und somit potenziell von den Konsequenzen des MFT-Positionspapiers betroffen gewesen wären [11–13]. Die Kernforderung des Positionspapieres des MFT lautete wie folgt:

„Zur Qualitätssicherung der Promotionen in der Medizin sieht der Medizinische Fakultätentag (MFT) die Notwendigkeit einer stärkeren Verankerung wissenschaftlicher Inhalte im Studium. Eine bereits im Studium begonnene Promotionsarbeit muss in einem strukturierten Programm mit einer mindestens neun Monate dauernden ausschließlichen Tätigkeit für die Forschung erfolgen. Erfolgreiche Promotionen sollen publiziert werden.“

Die Analyse der Promotionsordnungen belegt, dass wesentliche Forderungen des MFT-Positionspapiers von 2016 bislang nur partiell umgesetzt sind. Positiv zu vermerken sind flächendeckend verpflichtende Betreuungsvereinbarungen, Kurse zu guter wissenschaftlicher Praxis, methodische Schulungen sowie die Option kumulativer Dissertationen. Demgegenüber bleiben strukturierte Promotionsprogramme zumeist fakultativ; Vorgaben zu Annahme- und Prüfungsmodalitäten, Begutachtungsverfahren, Plagiatskontrollen und Kriterien der Prädikatsvergabe („summa cum laude“) variieren weiterhin erheblich. Dadurch schwankt das Gesamtniveau der Ordnungen zwischen 87 Punkten (Brandenburg) und 53 Punkten (Kiel, Magdeburg, Mainz), obgleich der Bundesdurchschnitt von 57,3 auf 68,4 Punkte gestiegen ist.

Dieser Befund illustriert eindrücklich das bestehende Spannungsverhältnis zwischen der verfassungsrechtlich geschützten Satzungsautonomie der Hochschulen und dem zunehmenden Drang zur Etablierung bundeseinheitlicher Mindeststandards im medizinischen Promotionswesen. Promotionsordnungen entstehen fakultätsintern, meist im Promotionsausschuss oder Fakultätsrat und spiegeln lokale Forschungsprofile sowie wissenschaftliche Traditionen wider. Die aus Art. 5 Abs. 3 GG abgeleitete Satzungsautonomie berechtigt wissenschaftliche Hochschulen dazu, eigenverantwortlich Promotionsordnungen festzulegen [14]. Auch der Berliner Verfassungsgerichtshof bestätigte 2004, dass staatliche Eingriffe nur eingeschränkt möglich sind [15], da das Promotionswesen zum Kernbereich akademischer Selbstverwaltung gehört.

Vor diesem Hintergrund bleibt es Aufgabe zukünftiger Reformvorschläge, Lösungen zu entwickeln, die das Spannungsverhältnis zwischen der verfassungsrechtlich geschützten akademischen Selbstverwaltung und dem Wunsch nach bundeseinheitlichen Mindeststandards noch differenzierter und ausgewogener berücksichtigen, um Transparenz, Vergleichbarkeit und Chancengleichheit im medizinischen Promotionswesen nachhaltig zu verbessern.

### Strukturelles Scoring und seine Grenzen für die Bewertung medizinischer Promotionen

Das Scoring nach Sorg et al. [4] ergibt nun eine Rangfolge. An erster Stelle befindet sich die Promotionsordnung der 2014 gegründeten staatlich anerkannten Medizinischen Hochschule Brandenburg. Dies kann durch die Tatsache erklärt werden, dass sich die 2019 verfasste Promotionsordnung, die gemeinsam mit der Universität Potsdam und der BTU Cottbus entwickelt wurde, in hohem Maße an dem Positionspapier des MFT orientierte. Wichtig ist jedoch hervorzuheben, dass eine hohe Punktanzahl nicht gleichbedeutend ist mit einer hohen Qualität der darunter hervorgegangenen Promotionsleistungen. So gibt der Score nach Sorg et al. keinen Aufschluss über die Qualität des betreuenden Personals und oder die Qualität der wissenschaftlichen Strukturen einer Fakultät. Einige medizinische Fakultäten im unteren Viertel des aktuellen Scorings gehören so nachweislich zu den forschungs- und publikationsstarken Standorten, dies muss kritisch reflektiert werden. Es ist dann naheliegend, dass Promovierende dort hochwertig betreut werden und die Promotionen eine hohe inhaltliche Qualität aufweisen können. Eine mögliche zukünftige systematische Analyse der Promotionsarbeiten (z. B. *Impact Factor von daraus resultierenden Publikationen*, jeweiliger Anteil publizierter Promotionen etc.) könnte Informationen darüber geben, ob gute Strukturvoraussetzungen auch zu wissenschaftlich guten Promotionen führen. Die Frage ist dann zu diskutieren, wie man diese definiert. Gute Strukturvoraussetzungen, insbesondere geforderte Fortbildungen in GWP und wissenschaftlichen Methoden, schaffen aber auf jeden Fall bessere und v. a. gleiche Voraussetzungen für die Promovierenden und sollten auch den Betreuern die Arbeit erleichtern. Wir sind der Meinung, dass medizinische Promotionen auch zu wissenschaftlichen Peer-reviewed-Publikationen führen sollten. Eine gute Methode, dies auch zu gewährleisten, stellen die kumulativen Promotionen dar, die weiterentwickelt und in allen Ordnungen klar adressiert und vielleicht auch favorisiert werden sollten. Um Freistellungen für die Promotionen zu ermöglichen, sollten alle Fakultäten Clinical Scientist-Programme anbieten und dies auch in den Promotionsordnungen verankern. Wichtig zu erwähnen ist, dass es oft detaillierte Ausführungsbestimmungen zu den Promotionsordnungen gibt, die aber nicht immer öffentlich zugänglich sind und sich so unserer Bewertung entzogen haben.

## Fazit

Wichtige Punkte des MFT-Positionspapiers wurden bislang nicht vollständig umgesetzt. Die Qualität der Promotionsordnungen in Deutschland hat sich in den letzten 9 Jahren trotzdem verbessert. Untersuchungen, wie sich die vom MFT geforderten Strukturvorgaben auf die Qualität und Anzahl von medizinischen Promotionen auswirken, gibt es bislang nicht.

## Supplementary Information

Zusätzliche Tab. 2: Darstellung der Score-Struktur nach Sorg et al.

## Data Availability

Die erhobenen Datensätze können auf begründete Anfrage in anonymisierter Form beim korrespondierenden Autor angefordert werden. Die Daten befinden sich auf einem Datenspeicher an der Klinik für Allgemein- und Viszeralchirurgie des Universitätsklinikums Brandenburg.
