# Supplementary material for: The doctoral regulations of the medical faculties in Germany: What has changed in the last 9 years?
Source: Chirurgie (Heidelb). 2025 Oct 2;97(6):473–9. [Article in German] doi: 10.1007/s00104-025-02387-9 (PMC13212792; doi:10.1007/s00104-025-02387-9)
Supplement: Supplementary file 1 — Zusätzliche Tab. 2: Darstellung der Score-Struktur nach Sorg et al. [file 104_2025_2387_MOESM1_ESM.pdf]

Die Promotionsordnungen der Medizinischen Fakultäten in Deutschland

Was hat sich in den letzten 9 Jahren verändert?

Tabelle 2: Darstellung der Score-Struktur nach Sorg et al.

Punkte	Bewertungsmaßstab
**Teil A – Voraussetzungen (0 – 24 Punkte)**	
**1: Annahmeverfahren**	
**0**	Keine näheren Angaben bzw. keine Anmeldung erforderlich
**4**	Anmeldung / Annahme des Dissertationsthemas bzw. des Promovenden oder Betreuungsvereinbarung / Doktorandenvertrag zwischen betreuendem Hochschullehrer und Promovend muss vorliegen
**8**	Betreuungsvereinbarung/ Doktorandenvertrag mit umfangreicher Beschreibung von Pflichten und Leistungen beider Unterzeichner muss vorliegen
**2: Gute wissenschaftliche Praxis**	
**0**	Keine schriftliche Verpflichtung auf die GWP bzw. die Teilnahme an einem Kurs oder Seminar zur GWP wird nicht vorausgesetzt; Einhaltung der GWP wird vorausgesetzt
**4**	Eine Verpflichtung des Promovenden auf die GWP wird gefordert und muss unterzeichnet werden
**8**	Die Einführung in die GWP durch Teilnahme an einem Kurs oder Seminar ist Pflicht
**3: Methodenkenntnis**	
**0**	Keine Angabe
**4**	Die Einführung in die Methodik durch den/die Betreuer/-in bzw. die Teilnahme an einem Kurs oder Seminar in Methodik ist fakultativ
**8**	Die Teilnahme an einem Kurs oder Seminar in Methodik ist Pflicht
**Teil B – Schriftliche Prüfungsleistung (0 – 26 Punkte)**	
**4: Wissenschaftlicher Beitrag**	
**0**	Keine Angaben zur Qualität der schriftlichen Promotionsleistung
**4**	Selbstständig verfasste und wissenschaftlichen Ansprüchen genügende Leistung
**8**	Beachtliche wissenschaftliche Leistung oder muss einen Beitrag zum Fortschritt der Wissenschaft darstellen
**5: Sprache der Dissertation**	
**0**	Keine Angaben zur Sprache
**3**	Dissertation, neben Deutsch und Englisch, auch in einer anderen Sprache möglich
**6**	Dissertation in Deutsch und Englisch möglich
**9**	Dissertation nur in Englisch möglich
**6: Kumulative Dissertation**	
**0**	Keine Angabe, nicht möglich
**3**	Möglich, aber keine genaueren Angaben zu den Voraussetzungen
**6**	Hinweise auf Autorenschaft (Erst- /Koautor
**9**	Hinweise auf Anzahl, Qualität des Journals und Autorenschaft
**Teil C – Mündliche Prüfungsleistung (0 – 24 Punkte)**	
**7: Thema der mündlichen Prüfung**	
**0**	Keine oder unzureichende Angaben über das Thema
**2**	Die schriftliche Promotionsarbeit
**4**	Die Dissertation sowie ihr zugehöriges Fachgebiet; vertraut mit der Breite des Fachgebietes der Dissertation

**6**	Neben der Dissertation und dem zugehörigen Fachgebiet auch angrenzende Fachgebiete
**8**	Die Dissertation und die gesamte Medizin
**8: Plenum der mündlichen Prüfung**	
**0**	Keine näheren Angaben zum Rahmen der mündlichen Prüfung
**4**	Geschlossener bzw. umschriebener Personenkreis (z. B. Prüfungskommission oder Promotionsausschuss)
**8**	Fakultätsoffene bzw. öffentliche Verteidigung
**9: Anteil der mündlichen Prüfungsleistung**	
**0**	Keine näheren Angaben zur erforderlichen mündlichen Leistung
**4**	Mündliche Leistung, ohne Angabe der Benotung der mündlichen Leistung bzw. keine Benotung vorgesehen
**8**	Die Benotung der mündlichen Leistung fließt in das Gesamtergebnis mit ein
**Teil D – Begutachtung und Bewertung (0 – 26 Punkte)**	
**10: Begutachtungsverfahren**	
**0**	Keine Begutachtung notwendig oder keine näheren Angaben
**2**	Begutachtung der Dissertationarbeit vorgesehen
**4**	Begutachtung ausschließlich durch interne Gutachter der Fakultät
**6**	Begutachtung durch interne Gutachter der Fakultät; ein externer Gutachter ist fakultativ
**8**	Begutachtung durch mehrere Gutachter, wobei mindestens ein Gutachter extern ist
**11: Plagiatsüberprüfung**	
**0**	Keine näheren Angaben bzw. keine Überprüfung auf ein Plagiat
4	Vereinzelter oder bei Verdacht auf Täuschung erfolgt eine Überprüfung auf ein Plagiat
**8**	Standardmäßige Überprüfung der Dissertationsarbeit (z. B. mittels Plagiatssoftware)
**12: Kriterien für die Bewertung summa cum laude**	
**0**	Keine oder unzureichende Angaben der Kriterien
**2**	Alle schriftlichen Gutachten und / oder Bewertungen der Disputation müsse mit den Noten und Prädikaten bewertet werden, welche noch zur Benotung summa cum laude führen, ohne weitere Leistungserbringung; zusätzliche Gutachten zur Promotionsarbeit sind einzuholen
**4**	Es müssen eine oder mehrere Publikationen in einer Fachzeitschrift als Koautor vorliegen (auch geteilte Erstautorenschaft möglich)
**6**	Es müssen eine oder mehrere Publikationen in einer Fachzeitschrift als Erstautor vorliegen (auch geteilte Erstautorenschaft möglich)
**8**	Es müssen eine oder mehrere Publikationen in einer Fachzeitschrift als Koautor vorliegen und die Bewertung der mündlichen Prüfung mit den Noten oder Prädikaten bewertet werden, welche noch zur Benotung summa cum laude führen
**10**	Es müssen eine oder mehrere Publikationen in einer Fachzeitschrift als Erstautor (auch geteilte Erstautorenschaft möglich) vorliegen und die Bewertung der mündlichen Prüfung mit den Noten oder Prädikaten bewertet werden, welche noch zur Benotung summa cum laude führen
